# Comparison of Th1/Th2 and Treg/Th17 ratios between wet and dry cupping therapies in Persian medicine 

**Published:** 2020

**Authors:** Reza Soleimani, Mojgan Mohammadi, Seyed Ahmad Saghebi, Ali Taghipour, Ali Khorsand Vakilzadeh, Jalil Tavakkol Afshari

**Affiliations:** 1 *Department of Persian Medicine, School of Persian and Complementary Medicine, Mashhad University of Medical Sciences, Mashhad, Iran*; 2 *Allergy Research Center, School of Medicine, Mashhad University of Medical Sciences, Mashhad, Iran*; 3 *Immunology Research Center, School of Medicine, Mashhad University of Medical Sciences, Mashhad, Iran*; 4 *Department of Internal Medicine, School of Medicine, Mashhad University of Medical Sciences, Mashhad, Iran*; 5 *Department of Epidemiology and Biostatistics, Health School, Mashhad University of Medical Sciences, Mashhad, Iran*; 6 *Department of Complementary and Chinese Medicine, School of Persian and Complementary medicine, Mashhad University of Medical Sciences, Mashhad, Iran*; 7 *Immunology Research Center, School of medicine, Mashhad University of Medical Sciences, Mashhad, Iran*; 8 *Vascular and Endovascular Surgery Research Center, Mashhad of Medical Sciences, Mashhad, Iran*

**Keywords:** Wet Cupping Therapy, Subsets of T lymphocyte, Persian medicine

## Abstract

**Objective:**

In Persian medicine (PM), wet-cupping therapy (WCT) is the most utilized approach. WCT is mostly done between the shoulders, which is referred to as “*hejamt-e-aam*” in the Persian language. CD4+T cells also refer to T helper lymphocytes play a critical role in the immune system. Naïve CD4+ T cells differentiate into at least four subsets, T helper 1 (Th1), T helper 2 (Th2), T helper 17 (Th17), and T regulatory (Treg) cells. The master regulator controlling each subset have been defined as follows, *Tbet* (Th1), *Gata3* (Th2), *RORγt* (Th17), *FoxP3* (Treg). The purpose of this study was to compare the effect of WCT and dry-cupping therapy (DCT) on the ratios of Th1/Th2 and Treg /Th17 in healthy individuals.

**Materials and Methods:**

Participants were divided randomly into two groups of 41 men in the WCT group and 40 men in the DCT group. Blood was taken, before, one and four weeks after the intervention. RNA was extracted from the peripheral blood mononuclear cells and the expression of *T-bet*, *GATA-3*, *RORγt*, and *Foxp3* genes were determined by using SYBR green RT-PCR technique.

**Results:**

The results showed that WCT increased the expression of*GATA-3*, *RORγt*, and *Foxp3 *transcription factor genes (p=0.009, p=0.001, and p=0.021, respectively). Although in the WCT group, the ratio of *Foxp3*/*RORγt *increased (p=0.048), but the ratio of *T-bet*/*GATA-3* (Th1/Th2) decreased (p=0.971).

**Conclusion:**

Our findings indicated that WCT may regulate the T subsets of lymphocyte and reduce inflammation.

## Introduction

There is an increasing appeal for traditional, complementary, and alternative medicine throughout the world (Mahdavi et al., 2012 ▶). In Persian medicine (PM), there are five methods of withdrawing blood: phlebotomy (“*Fasd*”), wet cupping (El Sayed et al., 2014 ▶; Kordafshari et al., 2015 ▶; Montazer and Namavary, 2016 ▶), leeching, ear stinging, and blood rising from the nose. Among all these approaches, "*hejamat*" (in Persian) or "*hijama*" (in Arabic), which means "sucking", is popular in different cultures in Europe and Eastern Asia with some differences in each region (El Sayed et al., 2014 ▶). 

Wet-cupping therapy (WCT), which is call “*hejamt-e-aam*”, is the most utilized approach of cupping in PM and it is mostly done between the shoulders butit can be also done on the head, legs, and waist. WCT is done by placing a plastic cup, which acts as a sucking tool, on the selected body part, and then the area is lacerated. Finally, the cup is placed on the lacerated area to drain the blood and interstitial fluids. The procedure should be repeated three to ﬁve times to drain the blood and fluids completely(El Sayed et al., 2014 ▶). Another cupping method is dry cupping therapy (DCT) where bloodletting is not involved.

Previous findings indicated that the interscapular site has special features due to its adjacent anatomical organs as well as the histological properties of the skin in that area. WCT has been used in PM for the treatment and prevention of various diseases (Kordafshari et al., 2015 ▶). Different characteristics of the cupping region of “*hejamat-e aam*” may contribute to its beneficial results. These features include the availability of brown adipose tissue or brown fat on the upper back of the chest and neck toward the shoulders( Yao et al., 2011 ▶), the proximity of the cupping region to the main vessel divisions carrying blood from the heart to the brain, the proximity to the sympathetic ganglia (stellate ganglion), and the thoracic duct. Based on Chinese medicine, the passage of ﬁve important channels of acupuncture, which can be stimulated by WCT, can release trapped energy (Wang and Wang, 2008 ▶) in the channels and help it ﬂow correctly (Kim et al., 2011 ▶). Several studies showed that WCT may have positive effects on blood high-density lipoproteins (HDL), low-density lipoprotein (LDL), and cholesterol. The researchers showed that WCT can reduce LDL and cholesterol even a month after treatment (Arslan et al., 2014 ▶; Zarei et al., 2012 ▶). The most-frequently reported positive results of cupping therapy is pain relief (Cao et al., 2015 ▶), as well as improving acne (Wang and Wang, 2008 ▶), herpetic lesions (Cao et al., 2010 ▶), coughing, asthma (Goodwin and McIvor, 2011 ▶), eczema (Yao and Li, 2007 ▶), migraines (Tabatabaee et al., 2014 ▶) and back pain (AlBedah et al., 2015 ▶). Another beneficial effect of WCT is the improvement of the quality of life. According to Kordafshari *et al.,* WCT can increase the quality of life one month after treatment (Kordafshari et al., 2017 ▶).

The immune system consists of two innate and adaptive components. Adaptive immunity is mediated by cells called lymphocytes, divided into two subsets of cellular and humoral (Abbas et al., 2015 ▶; Askari et al., 2016a ▶, 2016b ▶, 2018a,2018b; Askari and Shafiee-Nick , 2019a ▶,2019b ▶). T helper 1(Th1), T helper 2(Th2), T helper 17(Th17), and T regulatory (Treg) cells are all developed through differentiation of CD4+ T cells. The main function of Th1 cells is to activate macrophages. The most important cytokine produced by Th1 is interferon gamma (IFN-γ) which, along with interleukin 12 (IL-12), stimulates the differentiation of Th1 cells by activating transcription factors, *T-bet*, STAT4, and SATA1(Askari et al., 2016a ▶,2016b, 2018a,2018b; Askari and Shafiee-Nick, 2019a ▶, 2019b ▶; Kidd, 2003 ▶; Rahimi et al., 2017 ▶, 2018). Th2 cells, by secreting IgE, stimulate mast cells and eosinophils, which can eradicate parasitic infections. The cytokines produced by Th2 cells are interleukin 4 (IL-4), interleukin 5 (IL-5), and interleukin 13 (IL-13). IL-4 induces *GATA-3* gene expression. *GATA-3* is a transcription factor that acts as the most important trigger for the differentiation of Th2 (Boskabadi et al., 2018 ▶; Kidd, 2003 ▶; Rahimi et al., 2017 ▶,2018). Th17 cells recruit the leukocytes, especially neutrophils, into the infection site and IL-17is the most important cytokine produced by these cells. Th17 cell development depends on *RORγt* and STAT3 transcription factors. Th17 cells are an important component of the pathogenesis of many inflammatory diseases such as psoriasis, rheumatoid arthritis, inflammatory bowel disease, and multiple sclerosis (Abbas et al., 2015 ▶; Grant et al., 2015 ▶). Regulatory T lymphocytes (Treg) are subtypes of CD4+ T cells that suppress immune responses and maintain self-tolerance (Grant et al., 2015 ▶). Transforming growth factor-beta (TGF-β) and interleukin 10 (IL-10) are two important cytokines secreted by Treg cells. The activation of *Foxp3* transcription factor may result in the development and activation of these cells (Abbas et al., 2015 ▶; Askari et al., 2016a ▶,2016b,2018a,2018b; Askari and Shafiee-Nick, 2019a ▶, 2019b, Bacchetta et al., 2018 ▶).Several studies done using herbal medicine,evaluated changes in the ratios of lymphocyte cells subtypes and other cells involved in immunity; their finding suggested that cupping has a regulatory effect on T lymphocyte subsets (Askari et al.,2016 ▶, 2018a,2018b; Boskabady et al., 2016 ▶; Hashemzehi et al., 2016 ▶; Rahimi et al., 2018 ▶). The purpose of this study was to compare the effect of WCT and DCT on the ratios of Th1/Th2 and Treg /Th17 in healthy individuals.

## Materials and Methods

**Trial design and participants**

**Figure 1:**
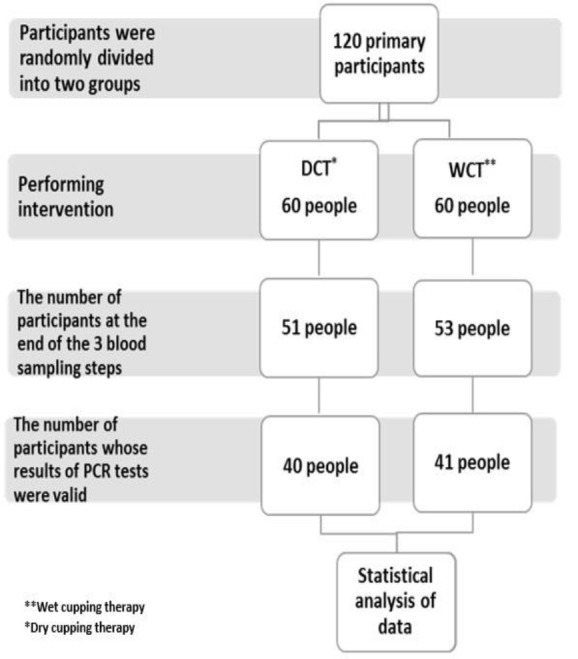
Consort flow diagram of the trial

This pre-post observational study investigated the effect of wet cupping on T cells subsets. The study subjects were selected from male patients who referred to the Persian Medicine clinic of the Mashhad University of Medical Sciences, Mashhad, Iran. The total number of subjects who participated in the study was 120. Participants were randomly divided into two groups using the random number table generated by computer. It was explained to each participant that he will be allocated in one of two different treatment groups, but they were not aware about the main intervention. The wet and dry cupping therapy groups consisted of 60 males. Information was taken from the records of the patients who attended the clinic in September 2017. From all subjects, 81 subjects who matched the inclusion and exclusion criteria, including 41 males in the WCT and 40 in the DCT groups, completed the study. The study process is shown in the consort flow diagram (Figure 1). The study was registered online at the Thai Clinical Trials Registry (registration No. TCTR20160609004) and Iranian Registry of Clinical Trials (registration No. IRCT20170806035515N2).

**Selection criteria**

**Inclusion criteria**

Inclusion criteria consisted of healthy men aged between 25 and40 years old, weighed above 50 kg and had a body mass index (BMI) of 20-30. All participants had a normal body temperature (37±0.5˚C) and blood pressure (130/85±10 mmHg for systolic and diastolic, respectively). All individuals confirmed that they did not have a history of chronic diseases such as diabetes, coronary and pulmonary disease, anemia, coagulation disorders, neurology and psychiatry disorder, severe infection, or allergy and they were not taking any anti-allergic medication. Applicants who were scored over 61 by the General Health Questionnaire-28(GHQ-28) and were overweight (BMI >30), were excluded from the study. 

**Exclusion criteria**

Exclusion criteria consisted of missing blood samples or becoming ill by any type of disease during the study.

**Intervention**

A verbal explanation was given to the eligible subjects, and then, their consent was obtained. All participants were examined by PM specialists. In the present study, the Persian subtitle of the GHQ-28, which was presented by Goldberg and Hiller, was employed(Goldberg and Hillier, 1979 ▶). The GHQ-28 was previously translated and approved in Iran by many studies (EBRAHIMI et al., 2007 ▶).

Before the intervention, 3 ml of venous blood was taken from each volunteer and placed in a tube (BD Co., England) with K3-EDTA anticoagulant in order to measure gene expression levels by real-time PCR method. For the WCT group, the area between the shoulders was disinfected by alcohol, and treated by oil, and slider cupping. Fixed cupping was performed on the "Aam" area and around it until the skin became expanded and reddened. Then, cups were exchanged with a 100- or 120-mlcup for 1 to 2 minutes with negative pressure cupping between the T2 and T4 on the backbones. Next, the cup was removed and seven scrapes in three rows (0.5 mm in depth) were applied by a scalpel (No. 22). Bloodletting was done about three times by applying a new disposable clean cup. In the end, the area of the procedure was cleaned using distilled water and dressed with honey. For the control DCT group, a similar procedure was used without skin scarification and bloodletting. The second blood sample (3 ml) was taken one week after the procedure. The same method was repeated during the fourth week after the procedure. Gene expression levels were measured using the SYBR green real-time PCR was done on both groups. For this purpose, the venous blood was used to extract the RNA and the cDNA was synthesized; then, transcription factor genes for T lymphocyte subsets, including *T-bet*, *GATA-3*, *Foxp3*, and *RORyt* using quantitative real-time PCR before the intervention and after one and four weeks.

**RT-PCR method**

Quantitative real-time PCR (qPCR) was conducted to examine the expression levels of the mRNA of the target genes in the Ficoll® isolated peripheral blood mononuclear cells (PBMCs) of the WCT group and DCT group according to the previous studies (Askari et al., 2018 ▶; Kianmehr et al., 2017 ▶), by the Roche LC96 real-time PCR system (Roche Biotechnology, Switzerland). The gene-specific primer for the qPCRs was designed based on the cDNA sequences of *T-bet*, *GATA-3*, *Foxp3*, and *RORγt* genes. The *GAPDH* gene was used as the internal control. The primer sequences used in the real-time PCR are shown in Table 1. The PCR SYBR® Premix Ex Taq (TliRNaseH Plus, Takara, Japan) was used according to the manufacturer's protocol. Total RNA was extracted from the PBMC using the Pars Tous Total RNA Extraction Kit (Pars Tous Biotechnology, Iran) according to the manufacturer’s protocol. The purity and concentration of total RNA were determined using a Nanodrop ND-2000 spectrophotometer (Thermo Electron Corporation, USA). The integrity of total RNA was checked by 1% agarose gel electrophoresis. cDNA was synthesized from 50µl of total RNA using the Yekta Tajhiz cDNA synthesis kit (YektaTajhizAzma, Iran) following the manufacturer’s protocol. The cDNA products were stored at -70°C to compare the gene expressions at a later time. No cell proliferation or stimulation was performed. The 2^-∆∆Ct^ method was chosen to calculate the relative mRNA expression levels of the target genes. Normalization was performed against GAPDH expression level in each group. After the mathematical calculations, the standard curve was plotted for each gene, and after ensuring the efficiency, the Ct value results were used in the 2^-∆∆Ct^ method for each sample. All data from the qPCRs were expressed as mean±standard error of the mean (mean±SEM). 

**Table 1:** The primer sequences used in the real-time PCR

**Gene**	**Primer**	**Sequence**
**GAPDH**	Forward	5'-CACTAGGCGCTCACTGTTCTC-3'
	Reverse	5'-CCAATACGACCAAATCCGTTGAC-3'
***T-bet***	Forward	5'-ATTGCCGTGACTGCCTACCAGA-3'
	Reverse	5'-GGAATTGACAGTTGGGTCCAGG-3'
***GATA-3***	Forward	5'-ACCACAACCACACTCTGGAGGA-3'
	Reverse	5'-TCGGTTTCTGGTCTGGATGCCT-3'
***Foxp3***	Forward	5'-GGCACAATGTCTCCTCCAGAGA-3'
	Reverse	5'-CAGATGAAGCCTTGGTCAGTGC-3'
***RORγt***	Forward	5'-CCCTGACAGAGATAGAGCACC-3'
	Reverse	5'-TTCCCACATCTCCCACATGG-3'

**Statistical analysis**

The calculated sample size was 40 for each group based on previous studies and the Cochran formula with 5% error. Baseline data were presented as the mean±standard error of the mean. All statistical parameters were analyzed using SPSS software (version 19.0). The level of statistical significance was set at p=0.05 for all analyses. The generalized estimating equations (GEE) method was used to analyze the differences in genes expressed in each group before the intervention and after one and four weeks. 

## Results

All participants' of this study were male with an average age of 31.86±6.28 years old in the WCT group and 33.61±6.4 years old in the DCT group. The minimum acceptable level for hemoglobin (HB) was 12.5 g/dl and for hematocrit (HCT) was 38%. Subjects with HB and HCT below the minimum level were excluded from the study. Table 2 shows the clinical characteristics of the participants. The mean values of these variables did not show a significant difference between the two groups. Table 3 shows the descriptive statistics of hemoglobin levels at three different stages. The statistical details related to the GEE method are shown in Table 4.

**Table 2:** Clinical characteristics of each group

	**WCT (mean±SD** ^*^ **)**	**DCT (mean±SD** ^*^ **)**
**Age**	32.90±6.28	33.80±6.4
**Height**	175.19±5.61	177.05±6.19
**Weight**	79.24±10.92	81.17±12.04
**BMI**	25.82±3.4	25.84±3.22

*Standard deviation

**Table 3:** The hemoglobin level in 3 different stages (mean±SD)

**Hemoglobin** **(g/dl)**	**Before intervention** **(mean±SD** ^*^ **)**		**One week after intervention** **(mean±SD** ^*^ **)**		**Four weeks after intervention** **(mean±SD** ^*^ **)**	
	WCT	DCT	WCT	DCT	WCT	DCT
	15.5±0.9	15.5±1	15.3±1.1	15.4±1	14.94±1	15.6±1

*Standard deviation

**Figure 2:**
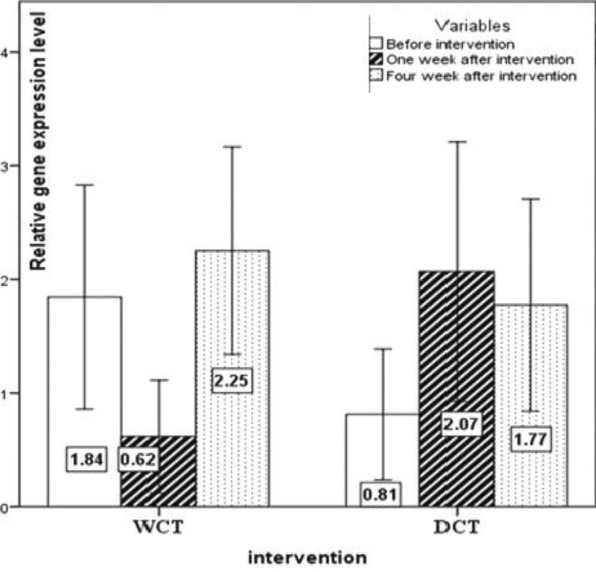
The expression pattern of the *T-bet* (Th1) transcription factor gene (mean±SEM)

**Figure 3:**
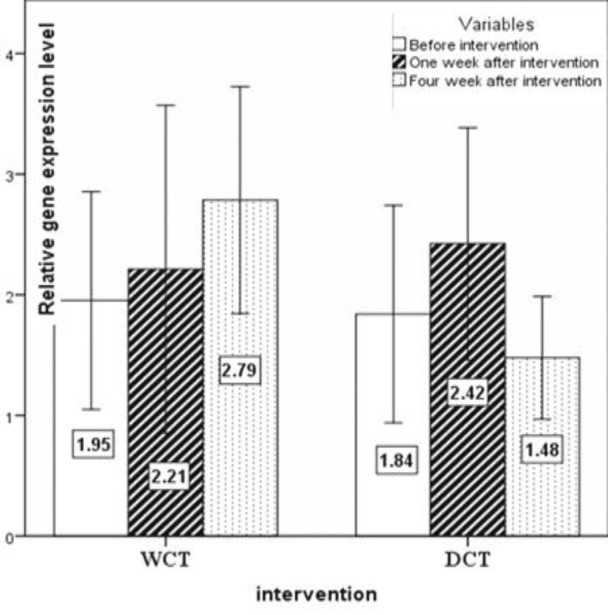
The expression pattern of the *GATA-3* (Th2) transcription factor gene (mean±SEM). p<0.05 WCT*Time

**Figure 4:**
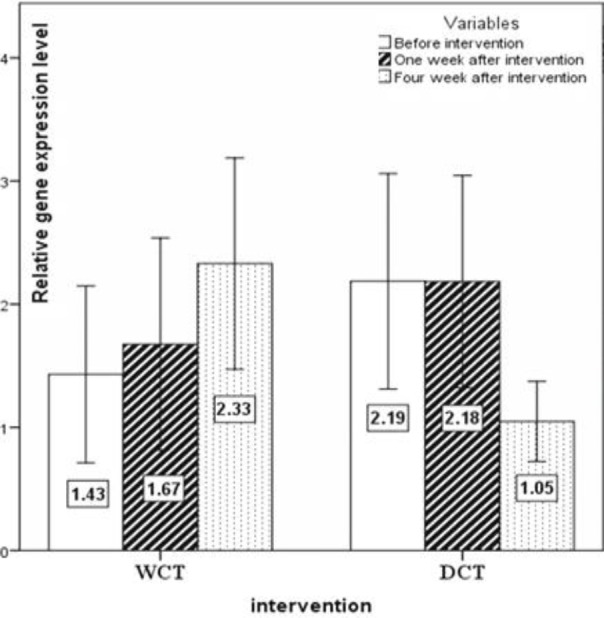
The expression pattern of the *RORγt* (Th17) transcription factor gene (mean±SEM). p<0.01 WCT*Time

**Figure 5:**
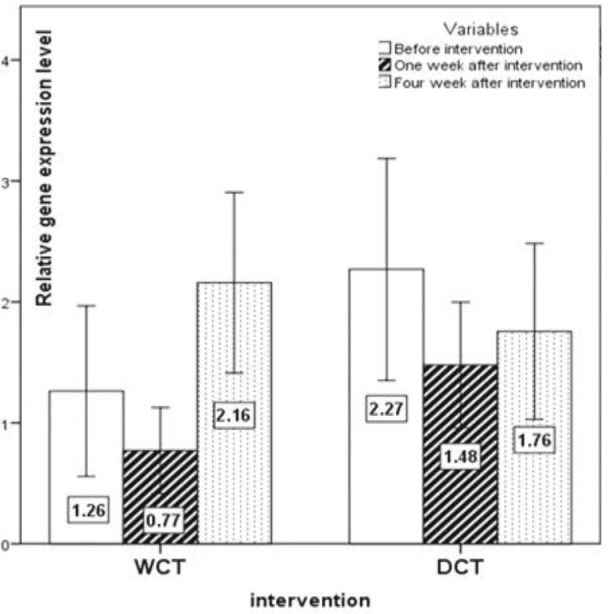
The expression pattern of the *Foxp-3* (Treg) transcription factor gene (mean±SEM). p<0.05 WCT*Time

The expression pattern of *T-bet*, *GATA-3*, *RORγt*, and *FoxP-3 *are shown in Figures 2, 3, 4 and 5, respectively. No significant changes showed by *T-bet* gene expression in both groups (Table 4). The expression levels of *GATA-3* gene significantly varied over time (p<0.05) (Table 4). As in the first week, increased expression was observed in both groups as compared to the baseline, but at the end of the fourth week the expression of *GATA-3 *increased in the WCT group and decreased in the DCT group. *RORγt *expression changed significantly over time in both groups (p<0.01) (Table 4). Expression of *RORγt *increased in the WCT but decreased in DCT groups. 

**Table 4:** The statistical details related to the GEE method

**Gene expression**	**Parameter**	**B**	**Std. Error**	**Wald ** **Chi-Square**	**df**	**p value** ^**^
***T-bet***	WCT	- 0.703	0.4737	2.206	1	0.137
	Time	0.003	0.0184	0.027	1	0.869
	WCT*Time	0.026	0.0257	1.058	1	0.304
***GATA-3***	WCT	- 0.975	0.5995	2.645	1	0.104
	Time	_ˉ_ 0.038	0.173	4.843	1	0.028^*^
	WCT*Time	0.068	0.0259	6.899	1	0.009^*^
***RORγt***	WCT	-1.103	0.4534	5.916	1	0.015^*^
	Time	-0.037	0.0136	7.529	1	0.006^*^
	WCT*Time	0.082	0.0196	17.680	1	0.00^*^
***Foxp3***	WCT	-1.350	0.4480	9.084	1	0.003^*^
	Time	-0.012	0.0203	0.357	1	0.550
	WCT*Time	0.061	0.0263	5.353	1	0.021^*^
***T-bet/ GATA-3***	WCT	0.219	3.3144	0.004	1	0.947
	Time	-0.010	1162	0.007	1	0.933
	WCT*Time	0.006	1678	0.001	1	0.971
***Foxp3/ RORγt***	WCT	-55.365	31.8656	3.019	1	0.082
	Time	-1.595	1.0378	2.363	1	0.124
	WCT*Time	3.341	1.6925	3.897	1	0.048^*^

*p values that have been underlined and in bold show significant changes.

**Association of the analyzed parameter for each group for four weeks.

The changes in *Foxp3 *expression level were not significant over time (p<0.05) (Table 4). There were significant differences between the two groups over time, as during the four weeks the mean gene expression for *GATA-3*, *RORγt*, and *Foxp3* in the WCT group were 0.068, 0.082, and 0.061 units more than the DCT group, respectively (p<0.001, p<0.001) (Table 4).

**Figure 6:**
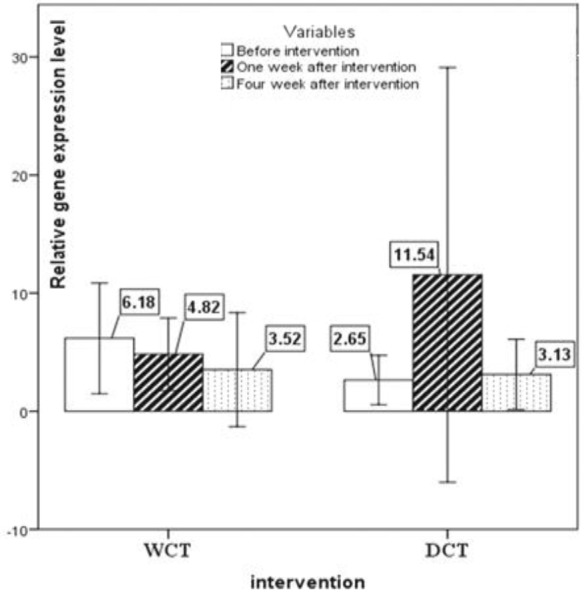
The gene expression ratio for*T-bet*/*GATA-3* (Th1/Th2) (mean±SEM)

**Figure 7:**
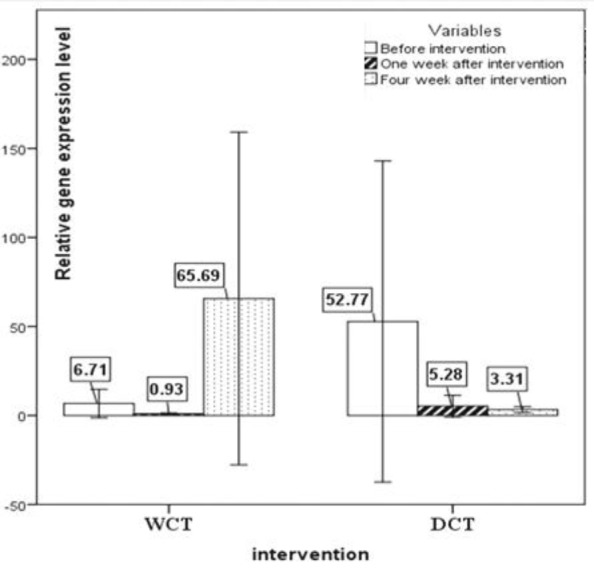
The gene expression ratio for the *Foxp-3*/*RORγt* (Treg/Th17) (mean±SEM). p<0.05 WCT*Time

*T-bet*/*GATA-3* and *Foxp-3*/*RORγt *gene expression ratio are shown in Figures 6 and 7, respectively. Though non-significantly, the *T-bet*/*GATA-3* ratio decreased in the WCT group but increased in the DCT group four weeks after the intervention. The ratio of *Foxp-3*/*RORγt*were not significantly change over time (p<0.05) (Table 4). However, there was a significant difference between the two groups over time (p=0.048) in terms of *Foxp-3*/*RORγt*ratio, as after the four-week process, *Foxp-3*/*RORγt* ratio in the WCT group was 3.341 units higher than that of the DCT group.

The expression of the *T-bet* gene correlated with the expression of the *GATA-3* in both groups before the intervention (p<0.05 and p<0.05); but, after the intervention, this correlation disappeared. In the WCT group, the expression of the *Foxp3 *gene had a positive relationship with *RORγt* gene expression in three time points of the study (p<0.001, R1=0.507, p<0.01, R2=0.680, p3<0.01, R3=0.327, respectively). In the DCT group, these two genes were positively correlated only in the pre-intervention stage (p<0.01, R=0.434).

## Discussion

As a commonly used procedure in PM,WCT is employed for treatment as well as prevention of different diseases (Refaat et al., 2014 ▶). Based on clinical findings, it is hypothesized that WCT boosts the immune system. Many studies reported the positive effects of WCT on different types of pain and some diseases. WCT is practiced based on ancient medicine, and recently, this traditional procedure is becoming more popular around the world especially in the Middle East. Although a number of researches have shown the efficacy of WCT in some diseases, more studies are needed to better understand its efficacy. Another beneficial factor making WCT popular is that the procedure is a nonchemical and non-pharmacological approach, and there are no reports of antagonistic effects of WCT when co-administered with drugs or pharmacological treatments (Mahmoud et al., 2013 ▶).

Kordafshari et al. study revealed that WCT can improve general health(Kordafshari et al., 2017 ▶). In a study done by Ahmed et al., topical cupping improved the clinical conditions of rheumatoid arthritis patients (Ahmed et al., 2005 ▶). El Sayedet al. study demonstrated that WCT may treat iron overload conditions in thalassemia (El Sayed et al., 2014 ▶). Another study done by Dons’koi et al., showed that DCT decreases the number and activity of the natural killer cells (NKc) (Dons' koi, et al. 2016 ▶). A recent review about cupping therapy mentioned that cupping may affect the immune system by inducing local inflammation, activating the complement system, and increasing the level of immune products such as interferons (Al-Bedah et al., 2018 ▶). In the first phase of this study, evaluation of the effect of WCT on the health level of patients and hematological factors showed improved health levels and decreased blood hematocrit and hemoglobin levels.

The main aim of our study was to investigate the effect of WCT on the unique transcription factors of T-lymphocyte subsets. The most important limitation of this study was lack of blindness and lack of control over external factors that may affect the immune system such as nutrition, physical activity, and mental status. The major findings of this study were increases in the *RORγt*, *Foxp3*, *GATA-3 *gene expression, and*Foxp-3*/*RORγt*gene expression ratio in the WCT group compared to the DCT group over time. *RORγt* is the transcription factor of Th17 cells; hence, it can be assumed that WCT may increase the number of Th17 cells leading to increased levels of cytokines such as IL-17 and IL-22. *Foxp3 *is the transcription factor of Treg cells; therefore, it can be assumed that WCT can increase the number of Treg cells and may have an effect on the production of Treg cytokines such as TGF-β and IL-10. Differentiation of CD4+ T cells to Th17 or Treg is mediated by TGF-β. In the presence of IL-6 or IL-21, CD4+ T cells differentiate into Th17 cells, and in the absence of IL-6, TGF-β drives differentiation into Treg cells (Lee, 2018 ▶). The Th17 cells, which are activated by specific inflammatory cytokine, may lead to tissue damages (Lubberts, 2010 ▶). The Treg/Th17 ratio is a major issue in the immunopathology of cancer and autoimmune diseases (Fasching et al., 2017 ▶). Increased Treg/Th17 ratio indicates that the immune system is more tolerant towards its own antigens and is less likely to develop autoimmune diseases. After developing cancer, an increase in Treg/Th17 ratio can lead to cancer metastasis(Maruyama et al., 2010 ▶). In this study, *Foxp-3*/*RORγt*gene expression ratio (Treg/Th17) in the WCT group was increased compared to the DCT group over time, which means WCT may have a positive regulatory effect on the immune system.

Although the *T-bet* changes were not significant, in the second week after the intervention the mean gene expression of *T-bet* was decreased in the WCT group. *T-bet* is the transcription factor of Th1 cells. Th1 produces inflammatory cytokine, meaning that WCT may decrease inflammation in the body. Moreover, *GATA-3* gene expression in the WCT group was higher than that of the DCT group following 4 weeks after the intervention. *GATA-3* is the transcription factor of Th2 cells. Therefore, it can be assumed that WCT may increase the number of Th2 cells and consequently, the production of Th2 cytokines such as IL-4, IL-5, and IL-13. The Th2 response is dominant in some diseases like asthma and allergy, but this is not true for many others. In WCT, the Th2 response is dominant, and it is correlated with the suppression of inflammation and down-regulation of the Th1 response. In many inflammatory diseases, Th1/Th2 response is dominant. The Th1/Th2 ratio is considered an indicator of inflammation in the body. Reducing this ratio indicates the suppression of inflammation in the body and health improvement. In this study, *T-bet*/*GATA-3*gene expression ratio (Th1/Th2) decreased in the WCT group, which means that WCT can be effective in the suppression of inflammation in some inflammatory diseases although this change was not significant.

Although some of these results were not statistically significant, the clinical findings of this study indicated that WCT can reduce itchiness, warmness, pain, and inflammation in patients. In PM, WCT is a method to purify and cleanse the body. This technique is especially used in treating diseases that are characterized by heat and inflammation. Warm inflammation is usually accompanied by redness, itching, burning, and warmness. Some autoimmune diseases like psoriasis, rheumatoid arthritis, and lupus erythematosus have some of these symptoms. Base on the findings of this study, WCT may be useful in treating or reducing symptoms of these diseases by increasing Th2 and Treg cells and decreasing Th1 and Th17.
